# Analysis of Neuropeptide S Receptor Gene (*NPSR1*) Polymorphism in Rheumatoid Arthritis

**DOI:** 10.1371/journal.pone.0009315

**Published:** 2010-02-22

**Authors:** Mauro D'Amato, Marco Zucchelli, Maria Seddighzadeh, Francesca Anedda, Staffan Lindblad, Juha Kere, Lars Alfredsson, Lars Klareskog, Leonid Padyukov

**Affiliations:** 1 Department of Biosciences and Nutrition, Karolinska Institutet, Stockholm, Sweden; 2 Department of Medicine, Karolinska Institutet, Stockholm, Sweden; 3 Institute of Neurogenetics and Neuropharmacology, CNR, Monserrato, Italy; 4 Institute of Environmental Medicine, Karolinska Institutet, Stockholm, Sweden; University of Wuerzburg, Germany

## Abstract

**Background:**

Polymorphism in the neuropeptide S receptor gene *NPSR1* is associated with asthma and inflammatory bowel disease. *NPSR1* is expressed in the brain, where it modulates anxiety and responses to stress, but also in other tissues and cell types including lymphocytes, the lungs, and the intestine, where it appears to be up-regulated in inflammation. We sought to determine whether genetic variability at the *NPSR1* locus influences the susceptibility and clinical manifestation of rheumatoid arthritis (RA).

**Methodology/Principal Findings:**

From the Epidemiological Investigation of Rheumatoid Arthritis (EIRA) case-control study, 1,888 rheumatoid arthritis patients and 888 controls were genotyped for 19 single-nucleotide polymorphisms (SNPs) spanning the entire *NPSR1* gene and 220 KB of DNA on chromosome 7p14. The association between individual genetic markers and their haplotypic combinations, respectively, and diagnosis of RA, presence of autoantibodies to citrullinated proteins (ACPA), and disease activity score based on 28 joints (DAS28) was tested. There was no association between diagnosis of RA and *NPSR1* variants. However, several associations of nominal significance were detected concerning susceptibility to ACPA-negative RA and disease activity measures (DAS28). Among these, the association of SNP rs324987 with ACPA-negative RA [(p = 0.004, OR = 0.674 (95% CI 0.512–0.888)] and that of SNP rs10263447 with DAS28 [p = 0.0002, OR = 0.380 (95% CI 0.227–0.635)] remained significant after correction for multiple comparisons.

**Conclusions/Significance:**

*NPSR1* polymorphism may be relevant to RA susceptibility and its clinical manifestation. Specific alleles at the *NPSR1* locus may represent common risk factors for chronic inflammatory diseases, including RA.

## Introduction

Rheumatoid arthritis (RA, OMIM 180300) is a complex disease with a broad spectrum of clinical manifestations, which presents with joint malfunction and pain, and general inflammatory symptoms in genetically predisposed individuals. Diagnosis is based on clinical consensus defined as the American College of Rheumatology (ACR) criteria.[1] Autoimmunity plays a key role in the development of RA, and recent progress in defining RA-specific autoantibodies has pointed to the involvement of different immunological pathways in this process, including B, T and macrophage cell functions. Remarkably, it has become evident that presence of autoantibodies to citrullinated proteins (ACPA) distinguishes two clinically relevant subgroups of RA: ACPA-positive and ACPA-negative. Although a differentiation between these two entities is not currently amenable on biological ground, it seems that ACPA-negative RA generally represents a milder disease with lower baseline activity, fewer erosions and better response to therapy. The heritability of RA was estimated from twin's studies between 40 and 77%.[2] Recently, significant progress has been made in our understanding of the genetic predisposition to RA, thanks to the implementation of genome-wide association (GWA) studies and intensive international collaboration. Together with a confirmation of the importance of *HLA-DRB1* shared epitope alleles and *PTPN22* gene variation, several other susceptibility loci were identified, and convincingly replicated in different Caucasian populations, including *TRAF1-C5*, *OLIG3-TNFAIP3*, *CD40*, *CCL21* and others.[3]–[6] While these results were obtained in RA cohorts mainly represented by ACPA-positive cases, fewer studies have reported genetic associations in ACPA-negative RA, namely with *HLA-DRB1*, *IRF5* and *DCIR* genes.[7]–[9] Overall, evidence is emerging of the existence of different genetic backgrounds and immune-response pathways contributing to the development of RA in ACPA-positive and ACPA-negative patients [10].

The neuropeptide S receptor gene (*NPSR1*), codes for a 7-transmembrane G protein-coupled receptor (GPCR) of poorly characterized function. Together with its ligand neuropeptide S (NPS), *NPSR1* is abundantly expressed in the brain, where it appears to play a role in stress, anxiety, and in the regulation of food intake.[11] However, *NPSR1* expression is increased during inflammation in several tissues, and recent work suggests that NPS-*NPSR1* signalling might be involved in the modulation of immune responses.[12]–[14] Genetic variation at this locus has been associated in different populations to asthma (OMIM 600807) and related traits and, more recently, also to inflammatory bowel disease (IBD, OMIM 266600).[13], [14] It has therefore been suggested that specific alleles at this locus might act as common genetic risk factors for the development of chronic inflammatory diseases. Here, using a candidate gene approach, we sought to determine whether genetic variation at the *NPSR1* locus influences individual predisposition to rheumatoid arthritis. For this purpose, 19 single nucleotide polymorphisms (SNPs), spanning the entire 220 kb region of the *NPSR1* gene, were selected based on reported associations and/or their tagging properties, and genotyped in 1808 RA cases and 888 healthy controls from the Epidemiological Investigation of Rheumatoid Arthritis (EIRA) study.

## Materials and Methods

### Study Population

The Epidemiological Investigation of Rheumatoid Arthritis (EIRA) is a population-based case-control study, which has been previously described by us.[15] EIRA cases are defined as individuals who received a new diagnosis of RA by their rheumatologist (in 85% of the cases within one year after first symptoms) and fulfilled the 1987 American College of Rheumatology (ACR) criteria for the classification of RA. ACPA status and disease activity score based on 28 joints (DAS28) were established at inclusion in the study, i.e. at first encounter with rheumatology specialist care, before any immunosuppressive therapy was initiated. EIRA controls are subjects randomly selected from the Swedish national population registry, and matched to cases based on subject's age, sex, and residential area. The ethics committee of Karolinska Institutet approved the study. For the purpose of this investigation, 1808 cases (1332 females, 476 males) and 888 controls (663 females, 225 males), >97% white Caucasians of Swedish origin, were included in the analyses. Data on ACPA and DAS28 at baseline were available, respectively, for 1308 (72.3%) and 629 (34.8%) of the patients included in this study.

### Ethics Statement

Verbal consent was received from all patients and it was registered at clinical journals according to Swedish law. Written consent was received from healthy control individuals. The ethics committee of Karolinska Institutet approved the study.

### Genotyping

Nineteen *NPSR1* SNPs were selected based on their reported associations with disease(s) and/or tagging properties (with Tagger software, http://www.broadinstitute.org/mpg/tagger/), and genotyped on genomic DNA using matrix-assisted laser desorption/ionization time-of-flight (MALDI-TOF) mass spectrometry and the iPLEX chemistry (SEQUENOM Inc.). Hardy-Weinberg Equilibrium (HWE) calculations were performed to verify that each marker was within allelic equilibrium in controls (cut-off p value = 0.05).

### Statistical Analysis

Statistical power was estimated using the package “genetics 1.2.1” from R (www.r-project.org). Data were simulated under a general multiplicative model with Risk Ratio between 1.05 and 2.00 and disease allele frequencies between 0.1 and 0.9. Disease penetrance was fixed to 50/1000000. SNP disease associations were tested with a logistic regression using a general additive model. More specific models (recessive/dominant/genotype) were applied to the SNPs showing association under the allelic model with significance lower than the nominal p = 0.05. Odds ratios (OR) were obtained by the model parameters and p values by a Wald test. Sex was inserted in the model as a covariate. To take into account multiple testing, the nominal significance threshold of p = 0.05 was corrected by finding the number of independent SNPs using Principal Component Analysis of the SNPs correlation matrix. Out of the 19 markers genotyped in this study, 11 resulted to be statistically independent, which fixed the significance threshold to p = 0.0045 after Bonferroni correction. Haplotypes were tested using “haplo.stats 1.3.0” from R. Here, haplotype inference is performed with a standard Expectation Maximization method and the association is tested with a Generalized Linear Model, which uses haplotypes posterior probabilities as weights. Haplotypes were tested over blocks of consecutive markers as defined in Haploview 4.1 (http://www.broad.mit.edu/mpg/haploview).

## Results

**Figure 1:**
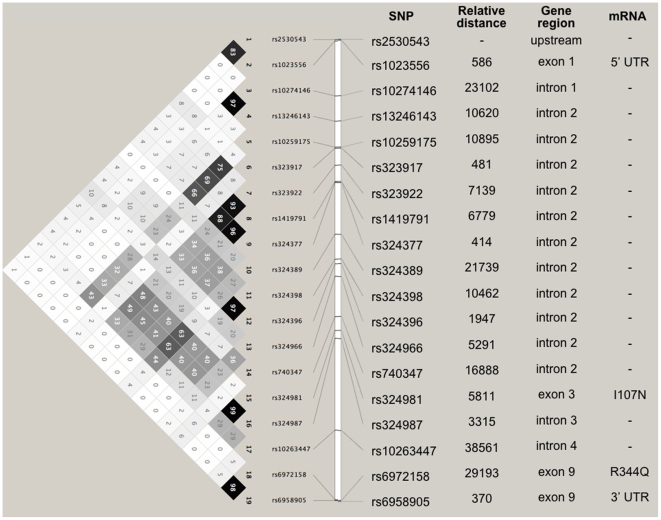
LD map and characteristics of the studied *NPSR1* SNPs. Left: LD and haplotype block structure obtained by Haploview 4 analysis of genotyping data from the EIRA population. The numbers in each box correspond to R-square values between SNPs. Right: SNPs are listed based on their relative distance in bp and their position in both the *NPSR1* gene and its corresponding mRNA).

Nineteen SNPs (dbSNPs rs2530543, rs1023556, rs10274146, rs13246143, rs10259175, rs323917, rs323922, rs1419791, rs324377, rs324389, rs324398, rs324396, rs324966, rs740347, rs324981, rs324987, rs10263447, rs6972158, and rs6958905), spanning approximately 200 kb of Chromosome 7p and including the entire *NPSR1* coding region, were genotyped on 2696 individuals from the EIRA study. The average success rate was 96.5%, and no SNP deviated from expected HWE. As shown in Figure 1, based on a Haploview 4.1 analysis of genotype data, these SNPs gave rise to linkage disequilibrium (LD) structure and minor allele frequencies (MAF) similar to those reported for other populations of European origin previously studied in asthma and IBD.[13], [14]

To test the potential contribution of the *NPSR1* locus to rheumatoid arthritis, we initially compared the allelic frequencies of each genotyped SNP in EIRA cases (n = 1808) and controls (n = 888). This analysis did not reveal any significant association (not shown). When available data on anti-citrullinated peptides antibodies were taken into account, however, sub-grouping of RA patients into ACPA-positive (n = 797) and ACPA-negative (n = 511) individuals provided evidence of an association regarding ACPA-negative RA for three markers, namely SNPs rs324389 [p = 0.008, OR = 0.802 (95% CI 0.681–0.944)], rs324981 [p = 0.026, OR = 0.837 (95% CI 0.716–0.979)] and rs324987 [p = 0.041, OR = 0.848 (95% CI 0.724–0.993)] (Table 1). In particular, the two latter SNPs appeared to exert a stronger predisposing effect under a recessive model, which withstood Bonferroni correction for multiple testing for rs324987 [p = 0.0044, OR = 0.674 (95% CI 0.512–0.888)].

**Table 1:** Association between *NPSR1* SNPs and rheumatoid arthritis in ACPA-negative patients vs controls.

	MAF§		statistics		
Marker#	Controls	Cases	p°	OR (95% CI)	best model
rs2530543 (T/c)	0.183	0.191	0.581	1.058 (0.867–1.292)	
rs1023556 (C/t)	0.214	0.221	0.676	1.040 (0.865–1.251)	
rs10274146 (A/g)	0.224	0.217	0.689	0.961 (0.791–1.168)	
rs13246143 (T/c)	0.214	0.209	0.744	0.969 (0.803–1.170)	
rs10259175 (A/g)	0.365	0.352	0.491	0.944 (0.802–1.111)	
rs323917(C/g)	0.069	0.059	0.276	0.840 (0.612–1.153)	
rs323922 (G/c)	0.433	0.412	0.279	0.918 (0.785–1.072)	
rs1419791 (G/a)	0.444	0.430	0.511	0.950 (0.816–1.106)	
rs324377 (C/a)	0.460	0.443	0.388	0.934 (0.800–1.090)	
**rs324389 (C/t)**	**0.390**	**0.340**	**0.008**	**0.802 (0.681–0.944)**	**allelic (p 0.008)**
rs324398 (C/g)	0.309	0.338	0.120	1.137 (0.967–1.337)	
rs324396 (C/t)	0.305	0.332	0.135	1.133 (0.962–1.333)	
rs324966 (G/a)	0.309	0.303	0.763	0.975 (0.827–1.150)	
rs740347 (G/c)	0.139	0.143	0.770	1.034 (0.827–1.292)	
**rs324981 (A/t)**	**0.490**	**0.446**	**0.026**	**0.837 (0.716–0.979)**	**recessive (p 0.006)**
**rs324987 (T/c)**	**0.490**	**0.450**	**0.041**	**0.848 (0.724–0.993)**	**recessive (p 0.004** * **)**
rs10263447 (G/c)	0.220	0.193	0.080	0.841 (0.693–1.022)	
rs6972158 (A/g)	0.337	0.347	0.586	1.046 (0.890–1.230)	
rs6958905 (T/c)	0.339	0.348	0.603	1.044 (0.889–1.225)	

# Minor allele in lower case.

§ MAF  =  minor allele frequency.

°Minor allele is the tested allele.

*significant after Bonferroni correction for multiple testing.

We then sought to determine whether *NPSR1* polymorphisms have a disease-modifying effect, by correlating individuals' genotypes with RA disease activity expressed as Disease Activity Score in 28 joints (DAS28) in 629 patients, for whom this information was available. As reported in Table 2, a number of SNPs showed association of nominal significance with the DAS28 score. Among these, the association of the SNP rs10263447 remained significant after Bonferroni correction when a dominant model was tested on the entire sample [p = 0.0002, OR = 0.380 (95% CI 0.227–0.635)], and was observed both in ACPA-positive [n = 393, p = 0.005, OR = 0.378 (95% CI 0.194–0.739)]) and ACPA-negative patients [n = 235, p = 0.038, OR = 0.407 (95% CI 0.176–0.946)]). No other associations were detected either with single markers or when *NPSR1* haplotypes were inferred from individual SNP genotypes, in RA as a whole or when patients were subdivided into phenotypic groups as described above (not shown). Finally, inclusion of gender as a covariate in the statistical analysis did not affect the genetic effect estimates of the observed associations, with the exception of marker rs2530543 that, in these test, resulted associated (withstanding Bonferroni correction) with DAS28 only in male patients [n = 164, recessive p = 0.0041, OR = 0.275 (95% CI 0.115–0.655)].

**Table 2:** Association between *NPSR1* SNPs and DAS28 in rheumatoid arthritis patients.

	mean DAS28§			statistics		
Marker#	AA	Aa	aa	p°	OR (95% CI)	best model
rs2530543 (T/c)	5.636	5.191	5.233	0.649	0.957 (0.794–1.155)	
rs1023556 (C/t)	5.266	5.201	5.393	0.916	0.990 (0.832–1.180)	
rs10274146 (A/g)	5.247	5.350	5.169	0.676	1.038 (0.871–1.239)	
rs13246143 (T/c)	5.020	5.348	5.210	0.802	0.978 (0.824–1.161)	
rs10259175 (A/g)	5.230	5.190	5.455	0.432	1.063 (0.912–1.241)	
rs323917(C/g)	5.219	5.398	5.423	0.271	1.182 (0.878–1.592)	
rs323922 (G/c)	5.415	5.199	5.216	0.296	0.925 (0.801–1.070)	
rs1419791 (G/a)	5.431	5.181	5.238	0.283	0.926 (0.805–1.065)	
rs324377 (C/a)	5.440	5.172	5.226	0.229	0.916 (0.795–1.056)	
**rs324389 (C/t)**	**5.166**	**5.213**	**5.605**	**0.032**	**1.180 (1.015–1.372)**	**recessive (p 0.021)**
rs324398 (C/g)	5.320	5.157	5.206	0.240	0.914 (0.787–1.062)	
rs324396 (C/t)	5.322	5.153	5.256	0.314	0.925 (0.796–1.076)	
rs324966 (G/a)	5.305	5.104	5.350	0.194	1.107 (0.949–1.292)	
**rs740347 (G/c)**	**5.069**	**5.056**	**5.313**	**0.046**	**1.237 (1.005–1.525)**	**recessive (p 0.036)**
rs324981 (A/t)	5.221	5.194	5.384	0.340	1.072 (0.929–1.238)	
rs324987 (T/c)	5.383	5.203	5.226	0.352	0.934 (0.810–1.078)	
**rs10263447 (G/c)**	**6.170**	**5.252**	**5.179**	**0.008**	**0.783 (0.655–0.937)**	**dominant (p 0.0002** * **)**
**rs6972158 (A/g)**	**5.400**	**5.127**	**5.107**	**0.014**	**0.824 (0.707–0.962)**	**dominant (p 0.012)**
**rs6958905 (T/c)**	**5.107**	**5.126**	**5.402**	**0.013**	**1.214 (1.042–1.417)**	**recessive (p 0.007)**

# Minor allele in lower case.

§ A  =  major allele: a  =  minor allele.

°Minor allele is the tested allele.

*significant after Bonferroni correction for multiple testing.

## Discussion

Using a candidate gene approach, we tested here for the first time the *NPSR1* gene for association with rheumatoid arthritis, and genotyped 19 *NPSR1* SNPs in a large cohort of 1808 RA patients and 888 controls from Sweden. We detected several associations of nominal significance, both regarding ACPA-negative RA and when *NPSR1* genotypes were correlated with disease activity expressed as DAS28 scores. In particular, the associations between SNP rs324987 and ACPA-negative RA (p = 0.0044), and SNP rs10263447 and DAS28 (p = 0.0002) remained significant after correction for the multiple tests performed.

In contrast to the case for ACPA-positive RA, very few genes have been previously identified that appear to predispose to ACPA-negative arthritis and, as mentioned, this may reflect the existence of a predisposing genetic background different from, or only partially overlapping with, the ACPA-positive form of RA. ACPA-negative RA is generally characterized by a less severe phenotype, and our observation that *NSPR1* also associates with DAS28 score is intriguing. It is in fact tempting to speculate that genetic variability at the *NPSR1* locus has indeed an overall impact on RA susceptibility by affecting the inflammatory process leading to the establishment and clinical manifestation of this condition. Of note, recent work suggests that NPS-*NPSR1* signalling might be involved in the modulation of inflammatory and anti-bacterial responses in T cells and macrophages.[12] NPS stimulation of epithelial cells stably transfected with *NPSR1* results in the induction of expression, among others, of the neuropeptide substance P (SP), the pro-inflammatory cytokine interleukin 8 (IL8), and the interleukin 6 receptor (IL6R), together with the alpha subunit common to the 4 pituitary gland hormones chorionic gonadotropin (CG), luteinizing hormone (LH), follicle stimulating hormone (FSH) and thyroid stimulating hormone (TSH).[16] Hence, similar to other neuropeptides and their receptors, *NPSR1* appears to play a dual role along the hypothalamic-pituitary-adrenal (HPA) axis and in inflammation, and polymorphisms affecting its expression or function might thus perturb physiological responses ultimately leading to chronic inflammation and RA.

*NPSR1* causative polymorphisms have not yet been conclusively identified and, although association with disease(s) has been replicated in different populations, there has not been always complete overlap of markers in previous associations with asthma and IBD. How genetic variability at this locus can influence disease predisposition is therefore still poorly understood. The *NPSR1* gene extends for 220 kb of genomic DNA on chromosome 7p14, and more than 1400 SNPs mapping within this region have been identified through sequencing of individuals of different ethnicity. The SNPs analyzed in this study have been selected based on previous associations and tagging properties, and are mostly contained within *NPSR1* intronic sequences. In particular, while an association signal of nominal significance is observed for several markers spread over half of the gene (110 kb from intron 2 through exon 9), best statistical evidence withstanding correction for multiple tests was obtained for SNPs rs324987 and rs10263447. These two SNPs were not tested in asthma or IBD in previous studies, where association signals came mainly from a marker haplotype in intron 2,[13], [14], and map respectively in *NPSR1* introns 3 and 4 (Figure 1). They may thus not impact on the functional properties of the receptor, and either relate to intragenic regulatory elements or represent LD proxies for other causative polymorphisms. Interestingly, the SNP rs324987 that associates with ACPA-negative RA in this study is in strong LD (r^2^ = 0.99) with the marker rs324981. The latter corresponds to a coding polymorphism (Asn107Ile) that affects *NPSR1* expression and NPS efficacy,[17], [18] and which has been linked to airway hyperresponsivenes in Chinese asthmatics.[19] However, this SNP only shows association of nominal significance with ACPA-negative RA in our study. Hence, although a predisposing role cannot be excluded until all associated SNPs have been functionally tested, true risk variants may need to be sought through an extended and comprehensive analysis of the *NPSR1* locus, both at the genetic and functional level. Ideally, this may be accomplished also taking into account sex differences in *NPSR1* signalling, since gender-specific effects on disease associations have been observed, though no information is available on the potential effect of hormones on receptor function and/or expression.

In summary, we have provided here initial evidence that *NPSR1* SNPs associate with ACPA-negative RA and disease activity. While key causative polymorphisms await definitive identification through replication studies and functional characterization, this and previous findings strongly suggest that *NPSR1* genetic variants may represent common risk factors for chronic inflammatory conditions such as RA, asthma and IBD. *NPSR1* holds the potential to be exploited as a pharmacological target when sufficient information on its function(s) is available, and future studies on this receptor may thus ultimately be of relevance to the development of novel therapeutic strategies for the treatment of these common diseases.
